# Dietary supplementation of Chinese herbal medicines enhances the immune response and resistance of rainbow trout (*Oncorhynchus mykiss*) to infectious hematopoietic necrosis virus

**DOI:** 10.3389/fvets.2024.1341920

**Published:** 2024-04-17

**Authors:** Qi Wang, Yucai Pan, Jinqiang Huang, Yongjuan Li, Shenji Wu, Lu Zhao, Tongzhen Sun, Yujun Kang, Zhe Liu

**Affiliations:** ^1^College of Animal Science and Technology, Gansu Agricultural University, Lanzhou, China; ^2^College of Science, Gansu Agricultural University, Lanzhou, China

**Keywords:** rainbow trout, Chinese herbal medicine mixture, antioxidant, immune response, IHNV

## Abstract

Rainbow trout is a widely farmed economical cold-water fish worldwide, but the prevalence of infectious hematopoietic necrosis virus (IHNV) presents a severe risk to the aquaculture industry, resulting in high mortality and huge economic losses. In this study, the impacts of different concentrations (0, 10, 20, and 30 g/kg) of Chinese herbal medicine mixture (CHMM) on the immune response and resistance of rainbow trout to IHNV infection were evaluated. The results show that CHMM noticeably increased (*P* < 0.05) T-SOD, CAT, AST, ALT, ACP, and AKP activities and decreased MDA content. *NF-*κ*B, TNF-*α, *IFN-*β, *IL-1*β, *JAK1, HSP70*, and *HSP90* expressions were significantly upregulated (*P* < 0.05) in all CHMMs, while *SOCS2* expression was downregulated (*P* < 0.05). Following infection with IHNV, feeding rainbow trout with varying amounts of CHMM resulted in noticeably increased (*P* < 0.05) T-SOD, ACP, and AKP activities and significantly decreased (*P* < 0.05) MDA content and AST and ALT activities. *TNF-*α, *IFN-*β, *IL-1*β, *HSP70*, and *HSP90* expressions were significantly upregulated (*P* < 0.05) in all CHMMs, while the expressions of *JAK1* and *SOCS2* were downregulated. The expression level of the IHNV *G* protein gene at a dosage of 20 g/kg was notably lower than that of the other CHMM feeding groups. This study provides a solid scientific basis for promoting CHMM as an immunostimulant for boosting antiviral immunity in rainbow trout.

## 1 Introduction

Aquaculture is a growing industry that provides important proteins and contributes to the production of nutrition (1). Rainbow trout (*Oncorhynchus mykiss*), belonging to the Salmonidae family, is native to North America and Russia's Pacific coast, and it is widely farmed as a cold-water fish in the global aquaculture industry (2). Intensive farming conditions have contributed to economic growth while also leading to complex aquatic environments that make rainbow trout susceptible to pathogens (3). Infectious hematopoietic necrosis virus (IHNV), which belongs to the genus *Novirhabdovirus* of the family Rhabdoviridae, is highly pathogenic and widely transmissible, causing not only high mortality in rainbow trout but also brings huge economic losses to the global aquaculture industry (4, 5). Vaccines have long been the main choice for preventing viral diseases, but their efficacy is limited by financial constraints. Relevant studies have shown that immunostimulants enhance immune responses in fish (6). The significance of using immunostimulants in boosting the immune system and assisting in disease prevention in fish is increasing (7).

Chinese herbal medicine (CHM) has been utilized as an immunostimulant in China for thousands of years, encompassing a diverse array of organic compounds such as alkaloids, polysaccharides, flavonoids, and organic acids. Additionally, CHM contains essential nutrients that include vitamins, amino acids, and minerals (8). The active ingredients mentioned have been extensively studied for their powerful immune-enhancing, antioxidant, and direct anti-pathogen properties in preventing and controlling viral, bacterial, parasitic, and fungal diseases in fish (9). Moreover, compared with chemical agents, CHM possesses the advantages of low price, fewer side effects, and less environmental pollution. Consequently, CHM is considered a viable alternative to certain synthetic compounds, such as antibiotics and chemicals. The application of CHM in aquatic organisms can significantly improve their feeding capacity, increase their metabolic rate, accelerate protein biosynthesis, enhance antioxidant enzyme activity, and ultimately enhance their immune and disease resistance (10).

The addition of a 1:1:1 mixture of *Astragalus membranaceus, Angelica sinensis*, and *Crataegus hupehens* to the feed of Nile tilapia significantly increased the levels of LZM, SOD, and CAT. Furthermore, the survival rate of Nile tilapia in the herbal mixture-administered group was 70% compared to 35% in the control group after *Streptococcus lactis*-free infestation. These results suggest that *Astragalus membranaceus, Angelica sinensis*, and *Crataegus hupehens* act synergistically to improve the immunity and disease resistance in Nile tilapia (11). The addition of an herbal mixture of *Astragalus, Angelica, Crataegus pinnatifida, Glycyrrhiza uralensis*, and *Lonicera japonica* to the diet of a hybrid grouper (*Epinephelus lanceolatus*♂ × *Epinephelus fuscoguttatus*♀) significantly increased AKP, ACP, T-SOD, and CAT activities in the fish (12). *Codonopsis pilosula, Atractylodes macrocephala, Poria cocos, Rehmannia glutinosa, Glycyrrhiza uralensis, Crataegus pinnatifida, Rhus chinensis, Gardenia jasminoides*, and *Zingiber officinale* enhanced non-specific immunity in turbot (13). Similar studies also showed that CHMM, consisting of 12 herbs, enhanced T-SOD activity and LZM content in European eel (14). In addition, diets supplemented with CHMM also significantly increased LZM content and CAT, ACP, and AKP activities in Japanese sea bass (15). Cai et al. found that CHMM of *Ziziphus jujuba*, Chinese yam, and *Astragalus* extracts administered to juvenile rainbow trout infected with *Vibrio parahaemolyticus* for 56 days significantly upregulated the transcript level of *HSP90* (16). All these results indicated that the CHMM exerted a beneficial synergistic effect and effectively improved the immunity of fish against diseases. Therefore, it is important to understand the effect of CHMM as an immune booster on the immunity and disease resistance of rainbow trout.

In fish, the liver plays a crucial role in numerous metabolic functions and physiological processes, including detoxification, nutrient metabolism, and biosynthesis (17). Additionally, the liver serves as an important immune organ in fish and can be infected with IHNV. To the best of our knowledge, there is currently limited research on how CHMM functions as an immunostimulant and how it affects the immune response and disease resistance in rainbow trout. This study examined the effects of varying the dietary concentrations of CHMM on the immune responses and resistance of rainbow trout to IHNV infection. This study will be the basis for promoting CHMM as an immunostimulant to improve the resistance of rainbow trout to pathogen infections.

## 2 Materials and methods

### 2.1 Experimental design

The rainbow trout weighed ~30 ± 0.5 g and were obtained from a trout farm in Yongjing County, Gansu Province, China. The rainbow trout were acclimated at the Aquatic Science Training Center of Gansu Agricultural University for 2 weeks, and all fish were fed a basal diet during rearing. In this study, we used the ratio of the previously studied herbal compound formulations as a reference (14–16). The 10 kinds of CHM used in this experiment (Table 1), all purchased from a local drugstore, were finely ground by using a 100-mesh sieve, and each CHM was uniformly mixed well according to the same mass ratio, dried under sterile control, and then precipitated and stored at 4°C until use. The composition of the feed of rainbow trout is based on the observations from the previous study (28). CHMM was added at a ratio of 0, 10, 20, and 30 g/kg. A total of 480 rainbow trout were placed in four groups in four tanks, with each tank comprising 30 fish. The control group (0 g/kg group) and the feeding groups (10, 20, and 30 g/kg) were set up. After acclimatization, the fish were fed at 3% of their body weight for 35 days. A circulating water system was used, with a 24-h uninterrupted oxygen supply, a water temperature of 12.0 ± 1.0°C, a pH value 7.3 ± 0.3, dissolved oxygen of 8.5 ± 0.5 mg/L, and an ammonia nitrogen concentration of < 0.1 mg/L. This experiment complies with institutional guidelines and protocols approved by the Animal Ethics Committee of Gansu Agricultural University (GSAU-Eth-AST-2021-004).

**Table 1:** Chinese herbal medicines and their function.

**Medicinal herbs**	**Plant part**	**Main compounds**	**Main function**	**References**
*Astragalus radix*	Roots	Flavonoids, isoflavones, polysaccharides, and saponins	Antiviral, antibacterial, immune system-enhancing, immunostimulatory, antioxidant, immunomodulatory, hepatoprotective, etc.	(18)
*Codonopsis pilosula*	Roots	Polysaccharides, lignans, alkaloids, flavonoids polyyne, and polyacetylene glycosides	Immunomodulatory, antioxidant, antiviral, anti-inflammatory, hepatoprotective, renoprotective effects, etc.	(19)
*Angelica sinensis*	Roots	Volatile oil, organic acid, vitamin A, and carotenoids	Anti-inflammatory, immune-boosting, etc.	(20)
*Glycyrrhiza uralensis*	Roots	Glycyrrhetinic acid, glycyrrhizin, licorice sweetener, flavonoids	Immunomodulatory, anti-inflammatory, antioxidant, antibacterial properties, etc.	(21)
*Ophiopogon japonicus*	Earthnut	Steroidal saponins, homoiso flavonoids, polysaccharides	Anti-inflammatory, antioxidant, antibacterial, immunomodulatory effects, etc.	(22)
*Poria cocos*	Sclerotium	Triterpenes polysaccharides	Anti-inflammatory, immunomodulatory properties, etc.	(23)
*Lonicera japonica*	Flower bud	Organic acids, flavones, iridoids, saponins	Anti-inflammatory, antiviral, hepatoprotective, antibacterial, antioxidant effects, etc.	(24)
*Isatidis radix*	Roots	Alkaloids, nucleosides, amino acids, organic acids, aldehydes, phenylpropanoids, and flavonoids	Antiviral, antibacterial, anti-inflammatory, anti-endotoxic, etc.	(25)
*Isatidis folium*	Leaves	Alkaloids, flavonoids, polysaccharides, organic acids, and glycosides	Antiviral, antiendotoxin, antibacterial, anti-inflammatory, immunomodulatory, antioxidant, hepatoprotective, etc.	(26)
*Crataegus hupehensis*	Fruits	Flavonoids, triterpene acids, proantho-cyanidins, organic acids	Cardiotonic, antiarrhythmic, antihypertensive, hypolipidemic, antioxidant, etc.	(27)

### 2.2 Challenge test

After feeding for 35 days, the study tested the impact of CHMM on IHNV-infected rainbow trout. Each rainbow trout received an injection of 100 μL of the cell culture medium containing 500 plaque-forming units of IHNV into the peritoneal cavity. The IHNV *G* protein gene was relatively quantified after the challenge (29). During the IHNV challenge, the feeding conditions were not changed in all groups.

### 2.3 Sample collection

On days 7, 21, and 35 of CHMM feeding and day 12 of infection of IHNV in rainbow trout, four rainbow trout were randomly selected from each group, a lethal dose of MS-222 (Sigma Aldrich Co., St. Louis, USA) was administered, and the liver tissue was then excised, put in sterile cryotubes, and quickly transported to liquid nitrogen for storage at −80°C, until further use.

### 2.4 Determination of antioxidant and immune parameters

First, the liver tissue stored at −80°C was crushed using a freeze mill, then nine times the volume of saline was added according to kit instructions, mixed well, and centrifuged at 4°C at 2,500 r/min for 10 min. Later, the supernatant liquor was collected as a tissue homogenate. According to the commercial kit instructions (Nanjing Jiancheng Institute of Biological Engineering, Nanjing, China), total superoxide dismutase (T-SOD), malondialdehyde (MDA), catalase (CAT), aspartate aminotransferase (AST), alanine aminotransferase (ALT), acid phosphatase (ACP), and alkaline phosphatase (AKP) activities were measured using a spectrophotometer or a microplate reader.

### 2.5 Detection of liver immune-related gene expression

Total RNA was extracted from rainbow trout liver using the RNA extraction kit (TIANGEN, Beijing, China). Electrophoresis by 1.0% agarose gel was used to assess RNA integrity. RNA purity and concentration were measured using a NanoDrop 2000 spectrophotometer (Thermo Scientific, Waltham, USA) with a 260:280 ratio between 1.8 and 2.0. RNA samples were treated with DNase to remove genomic DNA. Subsequently, cDNA was prepared using a reverse transcription (Accurate Biology, Changsha, China). Real-time PCR was performed according to the manufacturer's protocol (*n* = 3) on a LightCycler^®^480 II instrument (Roche, Basel, Switzerland). The related target genes include *NF-*κ*B, TNF-*α, *IL-1*β, *IFN-*β, *JAK1, SOCS2, HSP70, HSP90*, and the IHNV *G* protein gene. β-actin was selected as an internal reference gene to normalize the mRNA expression. The qRT-PCR reaction system consisted of 20.0 μL of SYBR^®^ Green qPCR Super Mix (2 × ), 10 μmol/L of forward and reverse primers, 7.0 μL of RNase-free water, and 1.0 μL of cDNA template. The real-time PCR conditions were as follows: 95°C for 30 s, followed by 40 cycles at 95°C for 5 s and 60°C for 30 s. The primers for each gene are listed in Table 2. Immunology-related gene expression levels were examined using the 2^−Δ*ΔCt*^ method.

**Table 2:** Primer sequences used in this study.

**Genes**	**GenBank No**.	**Primer**	**Sequence (5^′^ → 3^′^)**	**Amplicon (pb)**	**TM (°C)**
*NF-κB*	XM_021600117.2	F	CCACAGAACAAGCCGAGCAT	139	58.5
		R	GGTGTTCCCACTGTCGTCTACT		
*IL-1β*	NM_001124347.2	F	CGTCACATTGCCAACCTCATC	74	57.5
		R	CAGGTCCTTGTCCTTGAACTCG		
*TNF-α*	NM_001124357.1	F	ACCCACCATACATTGAAGCAGA	94	57.0
		R	GGTGTCAGCGGAAAGATTAGGA		
*IFN-β*	XM_021624608.2	F	TTCCCAGCCACCGCATTT	85	56.4
		R	CCACTTAGTAGGCAGGTCGTCAG		
*JAK1*	XM_036966729.1	F	GGCTGTGGCACCAGAACTAA	90	58.0
		R	TGTTGGGACCGCAGCATT		
*SOCS2*	XM_021616321.2	F	TCTCATCTTCGGGAGTCAGTGTT	93	59.5
		R	AGGAGGTAGCCCTGGTAGGAGAT		
*HSP70*	NM_001124228.1	F	CAGTCATCACAGTCCCTGCCTA	118	58.5
		R	CGTTCCCTGGACTTGCCTTT		
*HSP90*	XM_021612439.2	F	CCGTCATCACCAAGCACAAC	132	56.8
		R	TCTCTTTGACCCGTTTCTCCTC		
*G gene*	NC_001652.1	F	TCCTCTCGTTCCCGAAGTGT	74	59.6
		R	CCTCAAGACATTCCTCTCTGCTC		
*β-Actin*	NM_001124235.1	F	TGGGGCAGTATGGCTTGTATG	122	58.1
		R	CTCTGGCACCCTAATCACCTCT		

### 2.6 Statistical analysis

SPSS 25.0 software was used for all statistical analyses (IBM Corp., Armonk, USA). The Shapiro–Wilk and Levene's tests were used to examine normal distribution (*SW* > 0.05) and equality of variance (*P* > 0.05). A one-way ANOVA with Tukey's multiple comparison test was used to analyze multiple comparisons, and differences were considered statistically significant when the *p-*value was < 0.05. All experimental data are presented as the mean ± standard error (SE).

## 3 Results

### 3.1 Changes in immune parameters after feeding the CHMM

**Figure 1:**
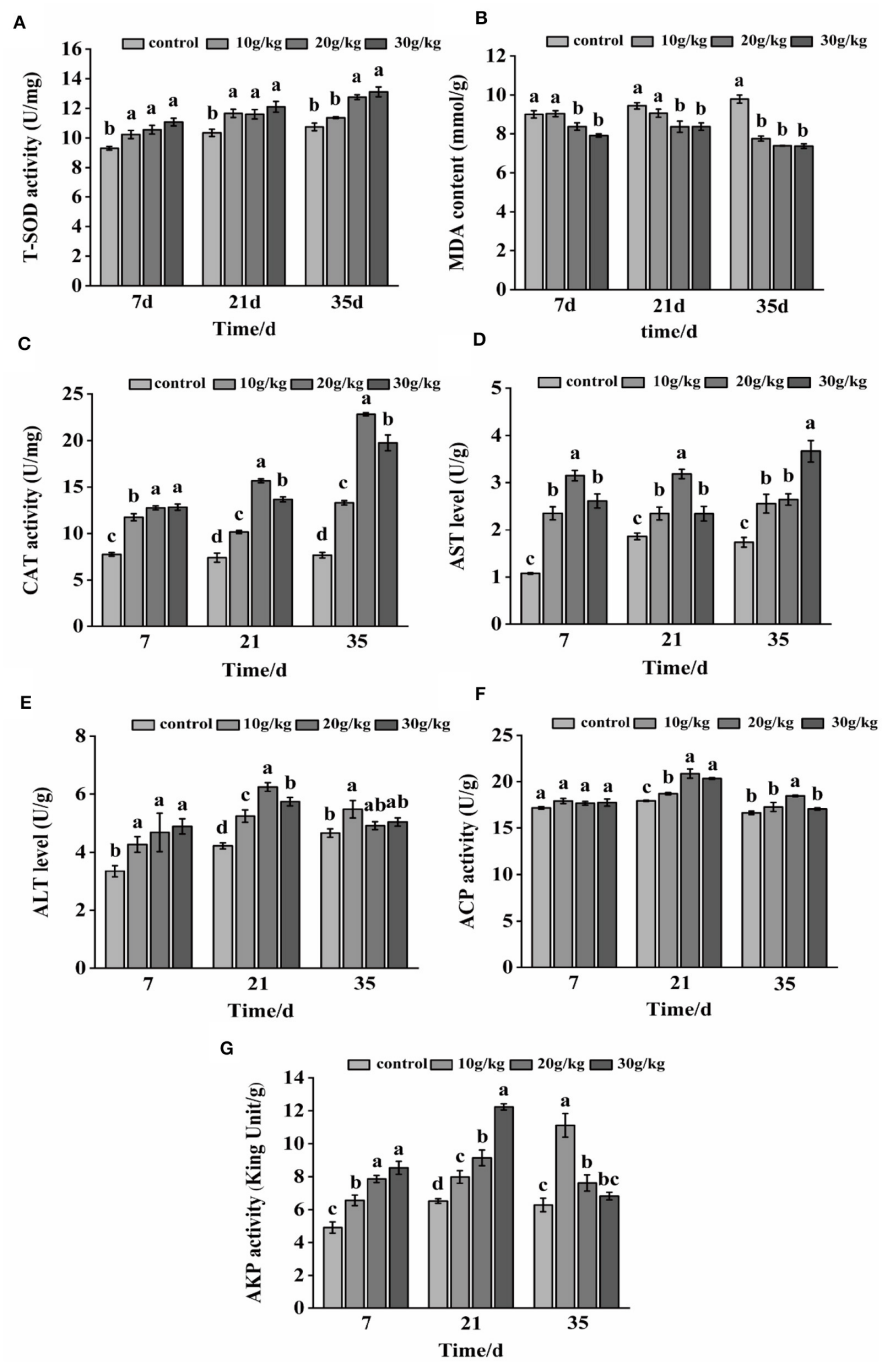
Effects of dietary CHMM on **(A)** T-SOD, **(B)** MDA, **(C)** CAT, **(D)** AST, **(E)** ALT, **(F)** ACP, and **(G)** AKP activities in the liver of rainbow trout; data are presented as mean ± S.E. with distinct superscript values denoting significance (*P* < 0.05); normal distribution *SW* > 0.05, homogeneity of variance *P* > 0.05.

During the whole feeding process of CHMM, liver T-SOD, CAT, AST, ALT, and AKP activities in each feeding group were found to noticeably increase (*P* < 0.05) during the feeding of CHMM as compared to the control group, as shown in Figure 1. At day 7, liver ACP activity did not noticeably increase (*P* > 0.05); however, all feeding groups showed a reduction in MDA content with an increase in CHMM dosage. At day 21, the MDA content decreased, with the lowest level at the 30 g/kg dosage, and CAT, AST, ALT, and ACP activities increased, reaching a maximum at the 20 g/kg dosage. The MDA content was noticeably decreased (*P* < 0.05) at day 35. Moreover, AKP activity at 10 g/kg, AST activity at 20 g/kg, and ACP activity at the 30 g/kg dosages were noticeably increased (*P* < 0.05) in rainbow trout compared to other CHMM feeding groups.

### 3.2 Changes in immune-related genes after feeding the CHMM

**Figure 2:**
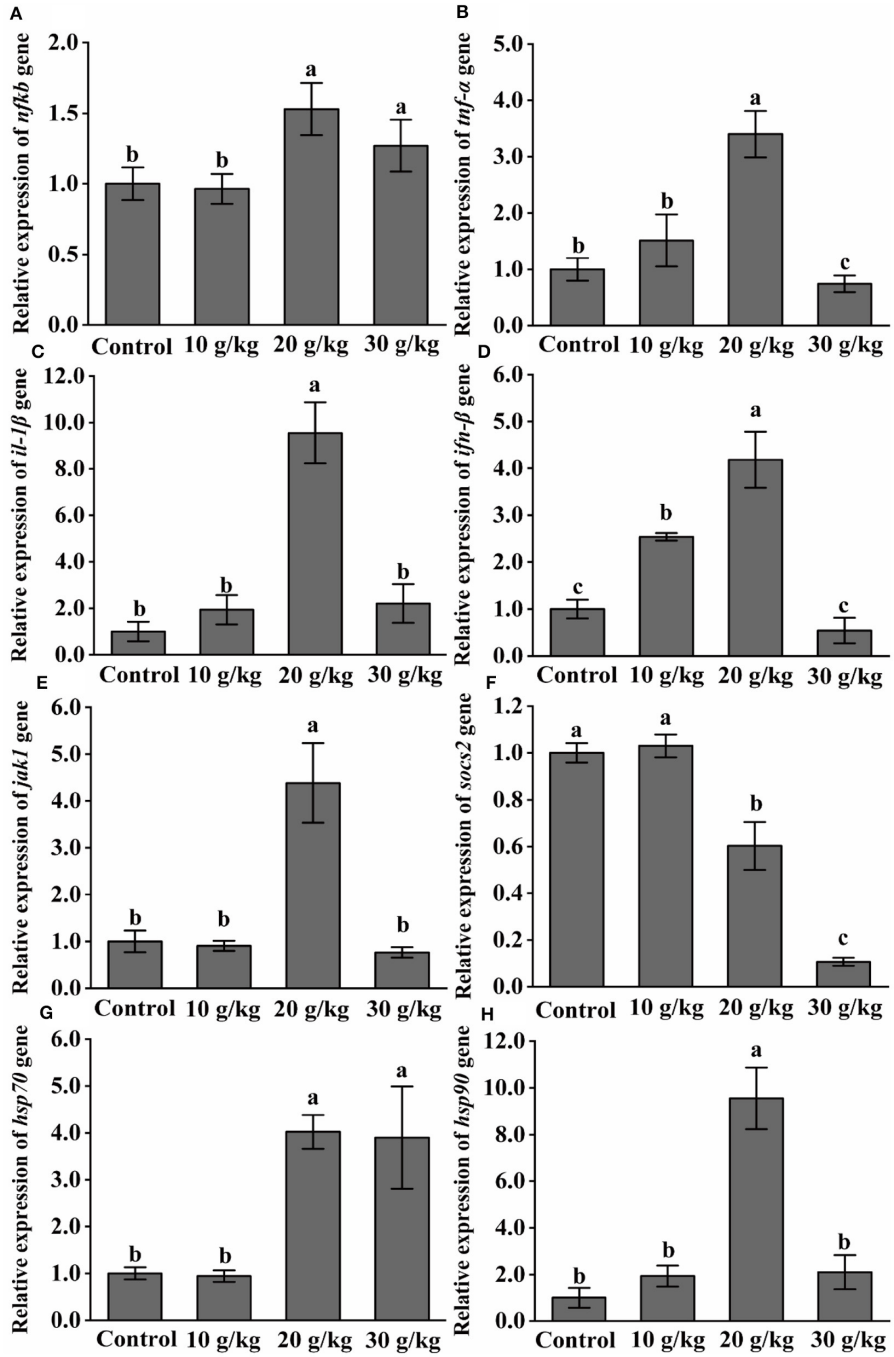
Effects of dietary CHMM on the expression of **(A)** *NF-*κ*B*, **(B)** *TNF-*α, **(C)** *IL-1*β, **(D)** *IFN-*β, **(E)** *JAK1*, **(F)** *SOCS2*, **(G)** *HSP70*, and **(H)** *HSP90* genes in the liver of rainbow trout; data are presented as mean ± S.E. with distinct superscript values denoting significance (*P* < 0.05); normal distribution *SW* > 0.05, homogeneity of variance *P* > 0.05.

As shown in Figure 2, at day 35, there was a noticeable upregulation (*P* < 0.05) of the *NF-*κ*B* expression level in the 20 and 30 g/kg feeding groups as compared to the control group. At day 35, all feeding groups had significantly higher (*P* < 0.05) levels of *IFN-*β and *TNF-*α, and the highest expression levels were observed in the 20 g/kg group. At day 35, the 20 g/kg group showed a significant upregulation (*P* < 0.05) of *IL-1*β, *JAK1, HSP70*, and *HSP90* expression levels. With an increase in dietary CHMM content at 35 days, *SOCS2* expression was significantly downregulated (*P* < 0.05), and the lowest expression was observed in the 30 g/kg group.

### 3.3 Changes in immune parameters following IHNV infection

**Figure 3:**
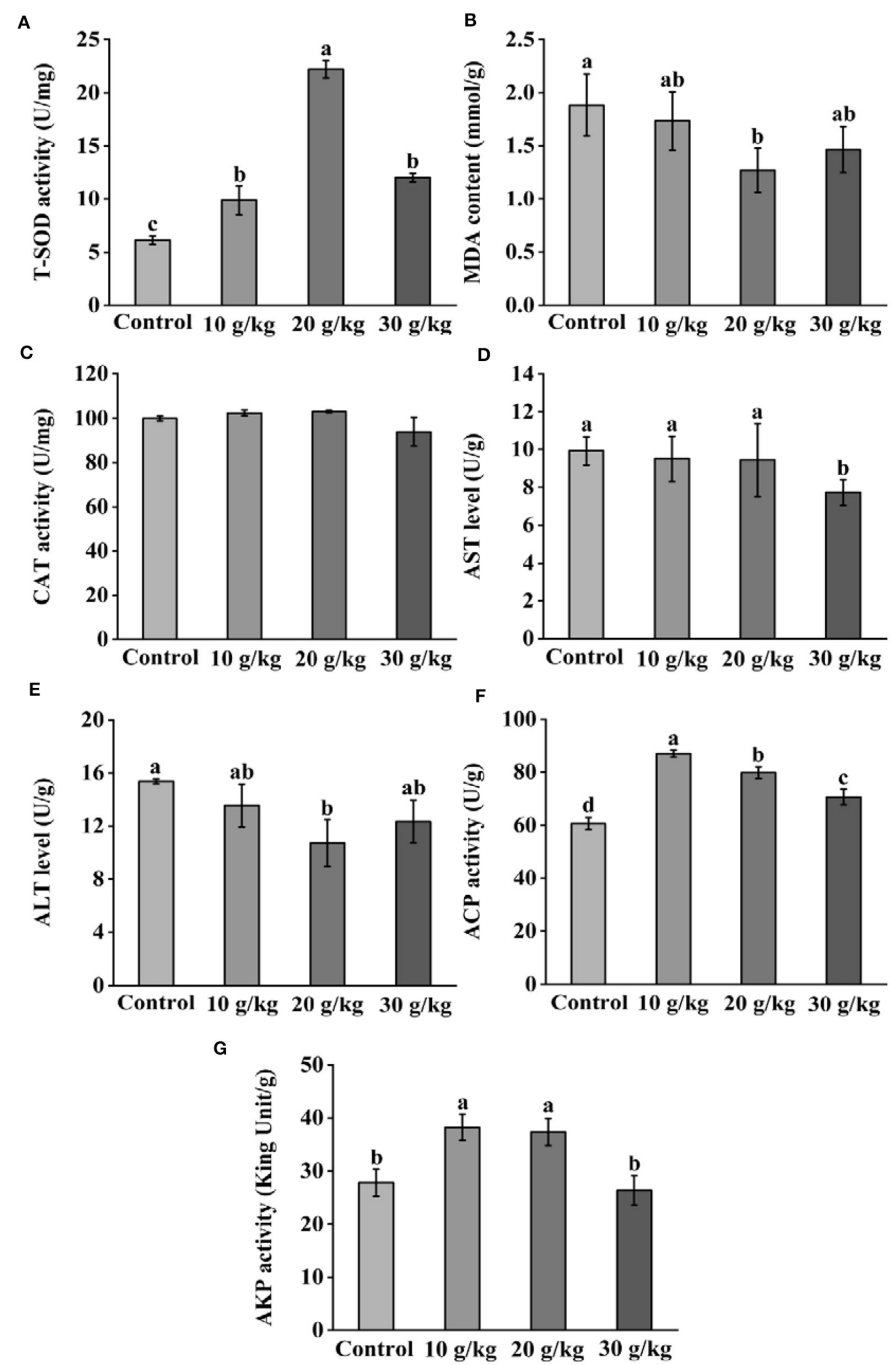
Effects of dietary CHMM on **(A)** T-SOD, **(B)** MDA, **(C)** CAT, **(D)** AST, **(E)** ALT, **(F)** ACP, and **(G)** AKP activities in the liver of rainbow trout after IHNV infection; data are presented as mean ± S.E. with distinct superscript values denoting significance (*P* < 0.05); normal distribution *SW* > 0.05; homogeneity of variance *P* > 0.05.

Following IHNV infection, all CHMM groups displayed noticeably higher (*P* < 0.05) ACP and AKP activities than the control group, as shown in Figure 3. Additionally, all CHMM groups showed a noticeable increase (*P* < 0.05) in liver T-SOD activity when compared to the control group; the highest level was observed in the 20 g/kg group. Additionally, compared to the control group, liver MDA content decreased and reached the lowest level in the 20 g/kg group. However, CAT activity did not noticeably change (*P* > 0.05) across all CHMM feeding groups. Furthermore, there was a decrease in liver AST, ALT, ACP, and AKP levels as the dietary CHMM levels increased among the three CHMM groups.

### 3.4 Changes in the expression of immune-related genes following IHNV infection

**Figure 4:**
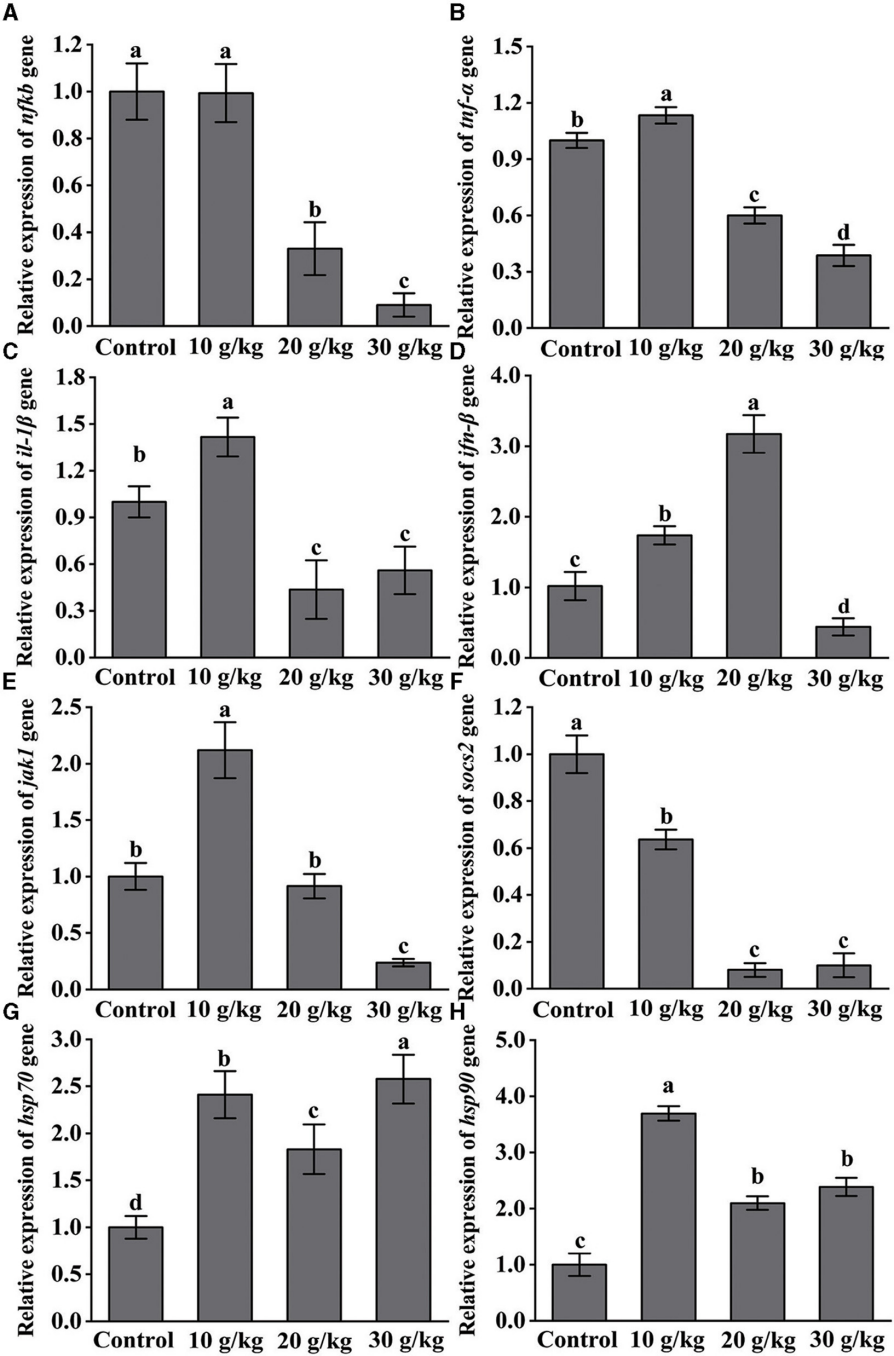
Effects of dietary CHMM on the expression of **(A)** *NF-*κ*B*, **(B)** *TNF-*α, **(C)** *IL-1*β, **(D)** *IFN-*β, **(E)** *JAK1*, **(F)** *SOCS2*, **(G)** *HSP70*, and **(H)** *HSP90* genes in the liver of rainbow trout after IHNV infection; data are presented as mean ± S.E. with distinct superscript values denoting significance (*P* < 0.05); normal distribution *SW* > 0.05; homogeneity of variance *P* > 0.05.

Considerably higher (*P* < 0.05) levels of *IL-1*β, *TNF-*α, *IFN-*β, *JAK1, HSP70*, and *HSP90* following IHNV infection in the 10 g/kg group were observed as compared to the control, as shown in Figure 4. In the 20 and 30 g/kg groups, there was a noticeable downregulation (*P* < 0.05) of the expression of *NF-*κ*B, TNF-*α, *IL-1*β, and *SOCS2*. Additionally as compared to the control group, the 10 g/kg group showed a significant upregulation (*P* < 0.05) of *JAK1* expression, which was then gradually lowered. Following infection, the amount of dietary CHMM was shown to significantly downregulate (*P* < 0.05) the level of *SOCS2*, and the lowest expression observed in the 30 g/kg group.

### 3.5 Disease resistance

**Figure 5:**
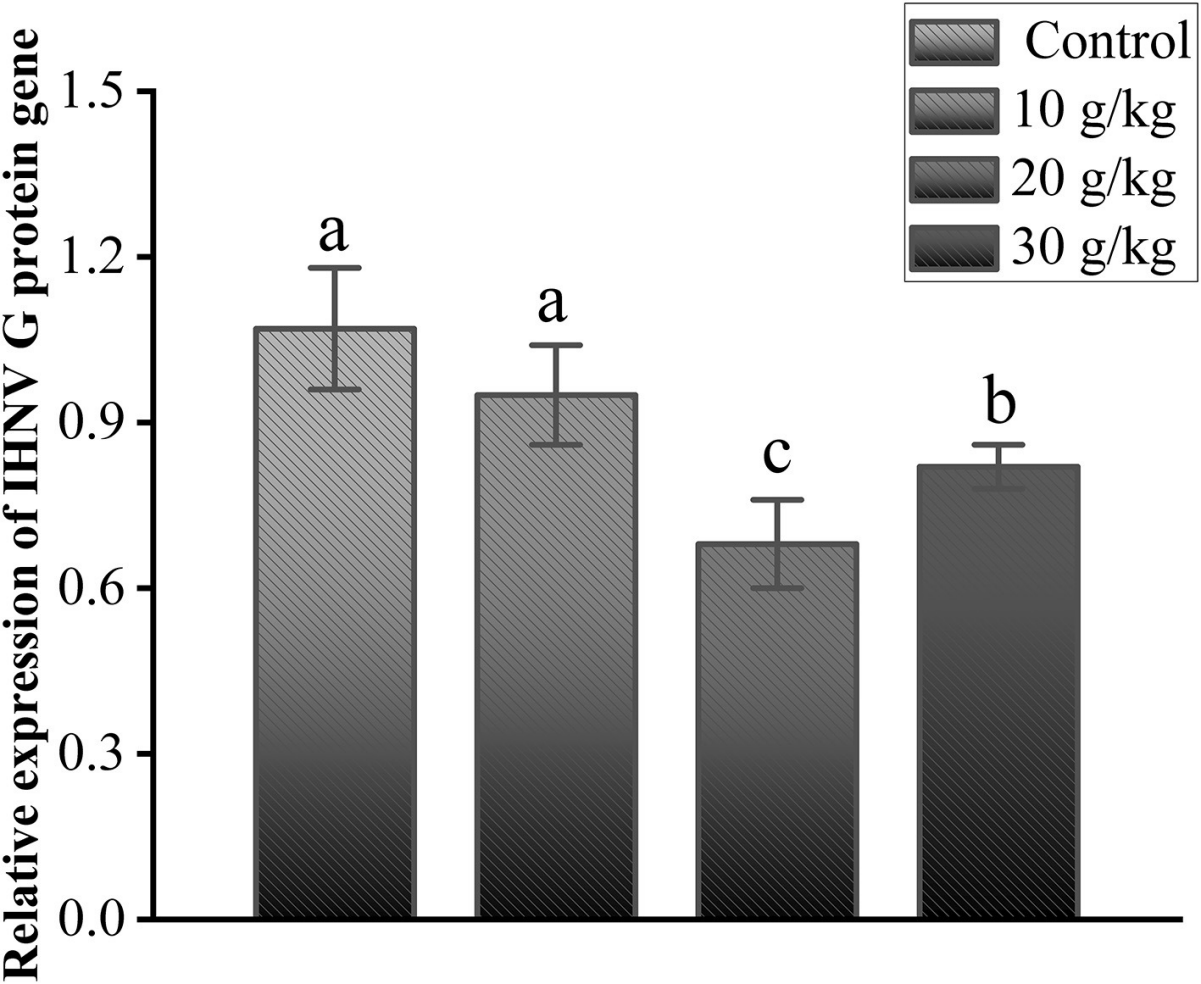
Effects of dietary CHMM on the expression of the IHNV G protein gene in the liver of rainbow trout after IHNV infection; data are presented as mean ± S.E. with distinct superscript values denoting significance (*P* < 0.05); normal distribution *SW* > 0.05; homogeneity of variance *P* > 0.05.

As shown in Figure 5, the relative expression of the IHNV *G* protein gene was analyzed compared to the control group, and no significant changes were observed in the 10 g/kg group; the expression level was significantly lower in the 20 g/kg and 30 g/kg groups (*P* < 0.05), and the 20 g/kg group had the lowest (*P* < 0.05) relative expression of the *G* protein gene. This finding further validates the significant role played by the dosage of 20 g/kg CHMM in enhancing immunity and anti-IHNV effects in rainbow trout.

## 4 Discussion

The outbreak of viral diseases has seriously hindered the sustainable development of rainbow trout farming and caused huge economic losses. Fortunately, CHMM has been demonstrated to have strong antiviral properties and is widely utilized in aquaculture. The impact of CHMM on the immunity and antiviral response in rainbow trout needs to be thoroughly understood. In this study, we examined the impact of feeding rainbow trout with varying levels of CHMM. Our focus was to evaluate antioxidant activity, immune responses, and resistance to IHNV infection in rainbow trout. The findings of this study are expected to provide valuable insights into the potential benefits of CHMM in promoting health and immunity in rainbow trout.

SOD and CAT play a vital role in the removal of reactive oxygen species derivatives and are regularly used as markers of oxidative stress (15, 30). MDA is considered a biomarker for assessing the levels of oxidative stress in various biological systems; MDA is toxic, and excessive levels of MDA can damage cell structure and function (31). CHM has been shown to have powerful antioxidant activity in earlier research (32). In this study, the administration of feeding diets containing CHMM drastically increased the SOD and CAT activities and notably decreased MDA content in rainbow trout. Similarly, the supplementation of Nile tilapia feed with a combination of Chinese herbs, including *Astragalus membranaceus, Angelica sinensis, and Crataegus hupehensis*, drastically increased SOD and CAT activities (11). Feeding a combination of Chinese herbs, including *Codonopsis pilosula, Astragalus, Medicago sativa, Panax notoginseng*, and goldenseal, had similar effects on European eel (14). Additionally, other research studies have shown that *Lonicera japonica, Radix isatidis, Astragalus radix, Glycyrrhiza uralensis Fisch, Angelica sinensis*, and hawthorn can also enhance T-SOD and CAT activities in animals (33). This study showed that CHMM may play a role in increasing the antioxidant capacity of rainbow trout. After IHNV infection, we observed a significant increase in T-SOD activity by feeding CHMM, but CAT activity had no significant change. The results suggest that the activity of liver T-SOD enzymes is relatively increased due to the manifold increase in the antioxidant capacity of rainbow trout in response to pathogen stimulation. However, other antioxidants that are part of the total antioxidant capacity of rainbow trout may inhibit CAT activity. Meanwhile, MDA content decreased, possibly because the body's increased antioxidant capacity reduced the production of lipid peroxidation products, resulting in a decrease in MDA content. In addition, the type of CHMM and the specificity of fish tissue can also lead to changes in antioxidant enzymes (16). In brief, the heightened activity of antioxidant enzymes improved the antioxidant capacity and inhibited the formation of free radicals in rainbow trout.

AST and ALT are important amino acid transferases and important markers of amino acid metabolism in fish (34). Earlier research has demonstrated that the administration of *Tridax procumbens* leaf extract to Nile tilapia could reduce serum ALT and AST levels (35); the addition of *Astragalus propinquus Schischkin* polysaccharides to the diet of *Channa argus* reduced serum AST and ALT levels (36). However, the findings of this study suggest that CHMM exhibits significantly elevated levels of AST and ALT enzymes in rainbow trout, suggesting that the effect of CHMM on the inhibition of amino acid metabolism may contribute to the observed increase in AST and ALT levels (37), and the antagonistic effect of CHMM may affect the normal metabolism of amino acids, but the specific mechanism is needed for further investigation.

ACP and AKP are crucial enzyme markers for immune function in fish. They play a key role in the immune response, helping to combat bacterial and pathogen infections as well as assisting in the removal of microorganisms and other foreign substances (38, 39). Previous research has shown that a Radix Rehmanniae Preparata polysaccharide diet in blunt snout bream significantly boosted ACP and AKP activities (40). Adding *Astragalus* polysaccharides to the diet of large yellow croakers had a similar effect (41). These results showed that ACP and AKP activities exhibited a significant increase as compared to the control group, which is consistent with previous investigations. After IHNV infection, CHMM had a significant impact on ACP and AKP activities in rainbow trout, suggesting that CHMM may enhance immunity in rainbow trout and similar effects have been observed in other organisms. For example, diets supplemented with *Achyranthes aspera* significantly increased serum AKP activity in *Labeo rohita* (42). Under conditions of *Aeromonas hydrophila* infection, the anthraquinone extract also increased serum AKP activity in *Megalobrama amblycephala* (43). These results indicated that feed supplementation with CHMM enhanced non-specific immunity in rainbow trout.

*NF-*κ*B* is a crucial transcription factor involved in the regulation of inflammatory responses. When activated, *NF-*κ*B* can trigger the expression of inflammatory factors, including *TNF-*α, *IL-1*β, and *IFN-*β (44). In this study, the levels of *NF-*κ*B, TNF-*α, and *IL-1*β were significantly altered in rainbow trout after 35 days of administration of different doses of CHMM. The findings suggest that CHMM may enhance the immune responses and disease resistance in rainbow trout. The results are consistent with those of previous studies demonstrating that fish fed with multi-fortified diets (e.g., *Ficus carica* polysaccharide, *Radix Rehmanniae Preparata* polysaccharide, and *Astragalus radix*) displayed higher levels of *IL-1*β and *TNF-*α than control fish (40, 45, 46). After IHNV infection, the expressions of *NF-*κ*B, TNF-*α, and *IL-1*β were significantly downregulated in all the groups of rainbow trout. Similarly, curcumin-based feed has a similar effect on grass carp with *Aeromonas hydrophila* infection (47). Thus, the downregulation of *NF-*κ*B, TNF-*α, and *IL-1*β expression levels suggests an enhancement of immunity in rainbow trout.

As the main antiviral component of the non-specific immune system with broad-spectrum antiviral effects, interferon (IFN) is essential for immunomodulation and antiviral effects (48). IFN is also a key modulator of the Janus kinase JAK/STAT pathway (49). JAK is a non-receptor tyrosine kinase located inside cells that is essential for several cytokine receptor-mediated signaling processes. The JAK/STAT pathway is considered one of the key pathways to JAK-mediated signaling in living organisms. The JAK/STAT pathway is activated by the binding of cytokines to the cytokine receptor on the surface of cell membranes and ultimately induces specific gene expression, which in turn promotes appropriate cellular responses (50). Furthermore, the JAK/STAT signaling pathway is regulated at different levels by different molecules and approaches, including a family of proteins called suppressor cytokine signaling (SOCS), which plays an essential role in specific and non-specific immunity through the negative feedback of cytokine signaling (51). In this study, CHMM significantly increased *JAK1* and *IFN-*β expressions, while the expression of S*OCS2* was decreased in rainbow trout. These results indicated that CHMM could enhance anti-inflammatory immunity in rainbow trout (52). After IHNV infection, in this study, the results showed that adding CHMM to the diet caused a significant upregulation of *IFN-*β expression, suggesting that feeding CHMM might enhance interferon induction and increase cell resistance to viral interference (53). The expression level of *JAK1* was significantly increased and then decreased compared to the control group. The results showed that CHMM may upregulate *JAK1* expression, and with an increase in the dose of CHMM, specific cell types and conditions may cause *JAK1* to be inhibited (54). Meanwhile, *SOCS2* expression was significantly diminished compared to that of the control. These findings showed that CHMM might activate the JAK/STAT pathway and enhance immunity in rainbow trout (55).

Heat shock proteins (HSPs) are a class of chaperone proteins that can be synthesized in large quantities in cells upon exposure to various environmental stressors. Previous studies have demonstrated that these proteins not only act as biomarkers but also play crucial roles in promoting cell survival, enhancing stress resistance, and facilitating tolerance to environmental pressures or injuries (56). *HSP70* and *HSP90*, belonging to the heat stress protein family, have been closely associated with organismal tolerance and survival in challenging environmental conditions. Early research has demonstrated that curcumin upregulates the expression of *HSP70* and *HSP90* in *Puntius sophore* (57). In this study, the CHMM noticeably increased *HSP70* and *HSP90* expression in rainbow trout, similar to previous findings. After IHNV infection, CHMM has also significantly upregulated *HSP70* and *HSP90* expression in rainbow trout. Similarly, a previous study showed that, under *Aeromonas hydrophila* infection, *Rheum officinale* Bail extract upregulated *HSP70* expression in *Megalobrama amblycephala* (58). Thus, these results suggest that upregulation of *HSP70* and *HSP90* expression might contribute to increased immunity and disease resistance in the organism. However, to elucidate the specific mechanisms involved, further studies are needed.

The IHNV G protein is a glycoprotein that plays an important role in IHNV pathogenicity and virulence, and since IHNV is an RNA virus, the IHNV *G* protein gene was quantitatively analyzed to determine the transcripts of the viral genes (59, 60). These results suggest that CHMM plays an important role in promoting immunity and protecting rainbow trout from IHNV infection, with the recommended dietary level of CHMM being approximately 20 g/kg.

## 5 Conclusion

In this study, the utilization of CHMM as an immunostimulant led to significant enhancements in antioxidant enzyme activities and non-specific immune parameters (T-SOD, MDA, CAT, AST, ALT, ACP, and AKP). Furthermore, the expression of immune-related genes (*NF-*κ*B, TNF-*α, *IFN*β, *IL-1*β, *JAK1, HSP70, HSP90*, and *SOCS2*) in rainbow trout were regulated, which ultimately improved the immune status of the organism and resistance to IHNV infection. In addition, the expression of the IHNV *G* protein gene at 20 g/kg was the lowest compared to that of the other CHMM feeding groups. Combined with the above findings, 20 g/kg CHMM supplemented in the diet had a significant protective effect against IHNV in rainbow trout. The findings of this research show that CHMM has an important function as an immunostimulant in aquaculture, which provides a scientific basis for improving antiviral immunity in rainbow trout.

## Data availability statement

The raw data supporting the conclusions of this article will be made available by the authors, without undue reservation.

## Ethics statement

The animal study was approved by the Animal Ethics Committee of Gansu Agricultural University. The study was conducted in accordance with the local legislation and institutional requirements.

## Author contributions

QW: Writing – review & editing, Data curation, Writing – original draft. YP: Writing – review & editing, Formal analysis, Methodology. JH: Writing – review & editing, Conceptualization, Funding acquisition, Project administration, Resources, Supervision. YL: Conceptualization, Project administration, Writing – review & editing. SW: Writing – review & editing. LZ: Writing – review & editing. TS: Writing – review & editing. YK: Writing – review & editing. ZL: Writing – review & editing.
